# Supplementary material for: Novel Driver Strength Index highlights important cancer genes in TCGA PanCanAtlas patients
Source: PeerJ. 2022 Aug 11;10:e13860. doi: 10.7717/peerj.13860 (PMC9375969; doi:10.7717/peerj.13860)
Supplement: Supplemental Information 2 [file peerj-10-13860-s002.zip › COHORTS/genes plots/cohorts/2021_8_16_14_9_distribution_cohorts_THCA.pdf]

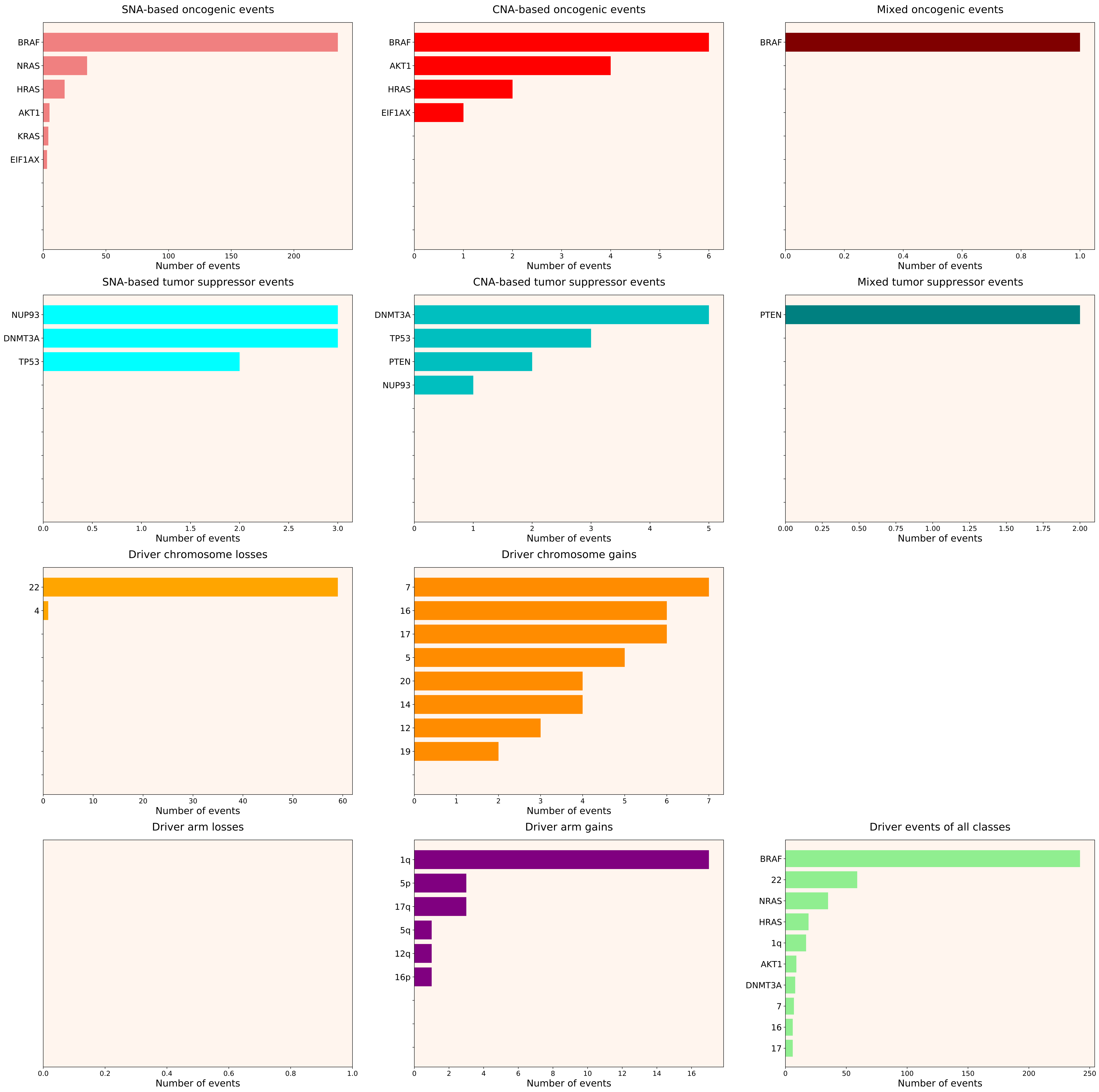
SNA-based oncogenic events
BRAF
NRAS
HRAS
AKT1
KRAS
EIF1AX
0 50 100 150 200
Number of events
CNA-based oncogenic events
BRAF
AKT1
HRAS
EIF1AX
0 1 2 3 4 5 6
Number of events
Mixed oncogenic events
BRAF
0.0 0.2 0.4 0.6 0.8 1.0
Number of events
SNA-based tumor suppressor events
NUP93
DNMT3A
TP53
0.0 0.5 1.0 1.5 2.0 2.5 3.0
Number of events
CNA-based tumor suppressor events
DNMT3A
TP53
PTEN
NUP93
0 1 2 3 4 5
Number of events
Mixed tumor suppressor events
PTEN
0.00 0.25 0.50 0.75 1.00 1.25 1.50 1.75 2.00
Number of events
Driver chromosome losses
22
4
0 10 20 30 40 50 60
Number of events
Driver chromosome gains
7
16
17
5
20
14
12
19
0 1 2 3 4 5 6 7
Number of events
Driver arm losses
0.0 0.2 0.4 0.6 0.8 1.0
Number of events
Driver arm gains
1q
5p
17q
5q
12q
16p
0 2 4 6 8 10 12 14 16
Number of events
Driver events of all classes
BRAF
22
NRAS
HRAS
1q
AKT1
DNMT3A
7
16
17
0 50 100 150 200 250
Number of events
